# Characterization of YABBY transcription factors in *Osmanthus fragrans* and functional analysis of *OfYABBY12* in floral scent formation and leaf morphology

**DOI:** 10.1186/s12870-024-05047-y

**Published:** 2024-06-21

**Authors:** Tingting Shi, Ling Zhou, Yunfang Ye, Xiulian Yang, Lianggui Wang, Yuanzheng Yue

**Affiliations:** 1 College of Landscape Architecture, Nanjing Forestry University, Nanjing, Jiangsu Province 210037 China; 2 Co-Innovation Center for Sustainable Forestry in Southern China, State Key Laboratory of Tree Genetics and Breeding, Nanjing Forestry University, Nanjing, 210037 China

**Keywords:** *Osmanthus fragrans*, *YABBY* gene family, Leaf development, Volatile organic compounds, β-ionone

## Abstract

**Background:**

The plant-specific YABBY transcription factor family plays important roles in plant growth and development, particularly leaf growth, floral organ formation, and secondary metabolite synthesis.

**Results:**

Here, we identified a total of 13 *OfYABBY* genes from the *Osmanthus fragrans* genome. These 13 *OfYABBY* genes were divided into five subfamilies through phylogenetic analysis, and genes in the same subfamily showed similar gene structures and conserved protein motifs. Gene duplication promoted the expansion of the *OfYABBY* family in *O*. *fragrans*. Tissue-specific expression analysis showed that the *OfYABBY* family was mainly expressed in *O*. *fragrans* leaves and floral organs. To better understand the role of *OfYABBY* genes in plant growth and development, *OfYABBY12* was selected for heterologous stable overexpression in tobacco, and *OfYABBY12*-overexpressing tobacco leaves released significantly fewer volatile organic compounds than wild-type tobacco leaves. Overexpression of *OfYABBY12* led to the downregulation of *NtCCD1/4* and decreased β-ionone biosynthesis. Correspondingly, a dual-luciferase assay showed that OfYABBY12 negatively regulated the expression of *OfCCD4*, which promotes β-ionone synthesis. Furthermore, tobacco leaves overexpressing *OfYABBY12* were curled and wrinkled and had significantly reduced leaf thickness and leaf inclusions and significantly extended flower pistils (styles).

**Conclusion:**

Overall, the results suggest that the *OfYABBY* gene family may influence the biosynthesis of the floral scent (especially β-ionone) in *O*. *fragrans* and may regulate leaf morphogenesis and lateral organs.

**Supplementary Information:**

The online version contains supplementary material available at 10.1186/s12870-024-05047-y.

## Introduction

*Osmanthus fragrans* is an important ornamental evergreen tree or shrub that is widely cultivated for its pleasing floral scent. It is commonly used in the landscaping and fragrance industries, where it has significant ornamental and economic value [1, 2]. Many *O*. *fragrans* cultivars have been cultivated in China for more than 2,500 years. These cultivars have significant differences in floral scent, floral color, leaf shape, and leaf color [3–5]. Previous studies have shown that the *YABBY* gene family not only participates in the formation and development of plant leaves and floral organs but also affects the biosynthesis of secondary metabolites, such as terpene floral metabolites [6–8]. In this study, it was hypothesized that the phenotypic difference in the leaves, flowers, and floral scents of different *O*. *fragrans* cultivars may be regulated by the *O*. *fragrans* YABBY family.

The YABBY family is a class of plant-specific transcription factors [9]. Due to the roles these transcription factors play in many of the biological processes of plants, the YABBY family has attracted great interest from researchers. The YABBY proteins contain two conserved domains, an N-terminal C_2_C_2_ zinc finger domain and a C-terminal YABBY domain. In *Arabidopsis thaliana*, *Solanum lycopersicum*, and other dicotyledonous plants, the YABBY transcription factors are classified into five subfamilies, namely CRABS CLAW (CRC), FILAMENTOUS FLOWER (FIL)/YABBY3 (YAB3), INNER NO OUTER (INO), YABBY2 (YAB2), and YABBY5 (YAB5). However, in *Oryza sativa*, *Zea mays*, and other monocotyledons, contain only four subfamilies and lack the YAB5 subfamily [9]. It has been indicated that the YABBY transcription factors in monocotyledonous and dicotyledonous plants have undergone functional divergence, and four gene duplication events have occurred in the YABBY family, leading to genes with innovative or redundant functions [10].

Some studies have shown that YABBY transcription factors are related to abaxial axis cell differentiation in lateral organs, thereby affecting leaf growth [11, 12] or the development of floral organs [13, 14] and fruit (seeds) [15, 16]. The YABBY family also participates in the biosynthesis of primary and secondary plant metabolites [8, 17, 18] and in response to biotic and abiotic stress [19–21]. In *A. thaliana*, *AtYAB2*, *AtFIL*, *AtYAB3*, and *AtYAB5* are expressed in the cotyledons, leaves, petals, stamens, and carpels and participate in regulating bud and leaf development and the formation of leaf polarity and floral organs [6, 7]. However, *AtCRC* is only expressed in nectaries and carpels, regulating the abaxial development of these structures [22]. Similarly, *AtINO* is only expressed in the abaxial axis of the outer integument and mainly regulates ovule development, thereby affecting seed growth and development [23, 24]. In *S. lycopersicum*, *SlYABBY1* regulates leaf and flower sizes [25], *SlYABBY2* regulates pericarp development and fruit maturation [26], and *SlCRCa* regulates flower and fruit size [27]. In *Chrysanthemum morifolium*, *CmDRP* (FlL/YAB3 subfamily) can regulate gibberellin biosynthesis to affect chrysanthemum plant height [28]. In addition, one study has shown that *YABBY* genes have dual functions, as they can act as both an activator and a repressor of secondary metabolites [29]. For example, *MsYABBY5* of *Mentha spicata* negatively regulates the synthesis of monoterpenes [8], whereas in *Artemisia annua*, *AaYABBY5* positively regulates the biosynthesis of artemisinin [17] and flavonoids [18]. Hence, YABBY transcription factors show tissue specificity in plants and play a variety of roles in plant growth and development.

Here, a comprehensive analysis of the *O*. *fragrans YABBY* gene family was conducted based on whole genome data, and *OfYABBY12* was stably overexpressed in tobacco to investigate whether *OfYABBY12* affects the growth and development of leaves and floral organs, as well as the synthesis of volatile organic compounds (VOCs). These results help to understand the OfYABBY family in *O*. *fragrans* and provide insight into the function of the *OfYABBY12* gene. The findings have important significance for further study of the functions of the OfYABBY family in *O*. *fragrans.*

## Materials and methods

### Plant materials

The experimental material was three-year-old *O. fragrans* ‘Rixiang Gui’ cuttings obtained from our *O*. *fragrans* germplasm resource bank (Jiangsu, China). Various tissues, including roots, stems, young leaves (from current-year branchlets), and mature leaves (from non-current-year branchlets) were sampled on March 26, 2022, and flowers at the full blooming stage were sampled on October 9, 2022. *Nicotiana benthamiana* and *Nicotiana tabacum* ‘K326’ seeds were saved by our group and planted in the greenhouse of Nanjing Forestry University under the following growth conditions: a 16/8 h light/dark cycle, a light intensity of 110 µmol photons m^− 2^ s^− 1^ white light, a temperature of 25 ± 2 °C, and 60–70% relative humidity.

### Identification of *O. fragrans* YABBY transcription factors and protein physicochemical analysis

The whole genome sequence of *O*. *fragrans* was obtained from the *O*. *fragrans* ‘Rixiang Gui’ genomic database [30]. HMMER software (v3.0) was then used to screen for family members in the Pfam YABBY (PF04690) domain [31]. Three online tools, Batch CD-search (http://www.ncbi.nlm.nih.gov/Structure/cdd/wrpsb.cgi), Pfam (http://pfam.xfam.org/search#searchBatchBlock), and SMART (http://smart.embl.de/smart/batch.pl), were used to validate the YABBY domains. In addition, ExPASy (https://web.expasy.org/compute_pi/) was employed to predict the molecular weight and isoelectric point of the identified YABBY protein sequences.

### Gene structure, conserved motif, and cis-acting element analysis

In this study, DNAMAN software (v6.0.40) was used for multi-sequence alignment and the analysis of the conserved protein domains of YABBY amino acid sequences in *O*. *fragrans*. In addition, MEME online software (http://meme-suite.org/) was used to analyze the conserved motifs of the OfYABBY protein sequences. The gene sequences 2,000 bp upstream of the start codon were obtained from the *O*. *fragrans* genome sequences, and the plantCARE database (http://bioinformatics.psb.ugent.be/webtools/plantcare/html/) was used to retrieve the obtained sequences to analyze the potential cis-acting elements in the promoter. Finally, TBtools software (v1.120) [32] was used to visualize the gene structure, conserved motifs, and cis-acting elements of OfYABBYs.

### Phylogenetic, chromosome localization, and gene duplication analysis

Genomic data were obtained from different databases, including *A*. *thaliana* (https://www.arabidopsis.org/), *S*. *lycopersicum* (https://solgenomics.net/), *O. sativa* (http://rice.uga.edu/), and *Z. mays* (https://maize-pangenome.gramene.org/). Furthermore, the protein sequences of YABBYs from *Vitis vinifera* [15], *Camellia sinensis* [33], *Mimulus lewisii* [34], *M*. *spicata* [8], *Chimonanthus praecox* [35], and *A*. *annua* [17] were obtained from relevant references.

Clustal X2.1 was used for the sequence alignment of the YABBY protein sequences of *O*. *fragrans* and other plants. In addition, MEGA X software was utilized to construct phylogenetic trees using the maximum likelihood method (Bootstrap = 1000) [36], and Fig Tree software (v1.4.3) was employed for visualization.

The chromosome locations of the *OfYABBY* genes were obtained based on the *O*. *fragrans* genome GFF3 file, and TBtools was used for visualization. Multiple Collinearity Scan Toolkits (MCScanX) and Ka/Ks calculators (NG) were used to further analyze the gene duplication events of the *OfYABBY* genes.

### RNA extraction and quantitative reverse-transcription polymerase chain reaction (qRT-PCR) analyses

The RNAprep Pure Plant Kit (Tiangen, Beijing, China) was used to obtain the total RNA of plant materials, and One-Step gDNA Removal and cDNA Synthesis Super Mix (Transgen, Beijing, China) were used for reverse transcription to obtain complementary DNA (cDNA).

Primer Premier 5.0 software was used to design the qRT-PCR primers (Table S1). The ABI StepOne Plus PCR system and SYBR® Premix Ex Taq™ II Kit (Takara, Dalian, China) were used to measure gene expression levels. In addition, 10-fold diluted cDNA was used as a template, and *OfACTIN* was used as an internal reference gene [37]. The 2^−ΔΔCT^ method [38] was employed to calculate the relative expression level. Biological triplicates were set up for each qRT-PCR reaction, and technical triplicates were set up for each biological triplicate.

### Subcellular localization and transactivation assay

The cDNAs of *OfYABBY3*, *OfYABBY5*, *OfYABBY7*, *OfYABBY8*, *OfYABBY12*, and *OfYABBY13* were cloned (primers shown in Table S2) and inserted into pCAMBIA1300 vectors to build the pCAMBIA1300-GFP-OfYABBY constructs. These constructed plasmids were transformed into *N*. *benthamiana* leaves by *Agrobacterium tumefaciens* strain GV3101 (Weidi Biotechnology, Shanghai, China). After 48 h, a Zeiss LSM 710 confocal microscope (Zeiss, Oberkochen, Germany) was used to observe fluorescence signals.

The cDNAs of *OfYABBY3*, *OfYABBY5*, *OfYABBY7*, *OfYABBY8*, *OfYABBY12*, and *OfYABBY13* were cloned into the pGBKT7 vector (primers shown in Table S2). These recombination vectors and the pGBKT7 empty vector were transformed into *Saccharomyces cerevisiae* (AH109). The suspended yeast was then diluted to different concentrations, and 5 µL dilutions were plated on SD-Trp, SD-Trp/Ade, and SD-Trp/Ade (X-α-gal) culture media. These were cultured at 30 °C in the dark for 3 d, and the growth status was then observed.

### Transformation of OfYABBY12 in N. tabacum

*A. tumefaciens* cells carrying the pCAMBIA1300-OfYABBY12 plasmid were cultured overnight, and the culture products were collected when the OD600 reached 0.4–0.5. A solution of 10 mM MgCl_2_, 10 mM MES, and 150 mM acetosyringone was used to resuspend the *Agrobacterium* cultures for transformation. In addition, tobacco (*N*. *tabacum* ‘K326’) seeds were sterilized and sown on MS medium, and when grown to three to four leaves, sterile tobacco leaves were cut into 0.5 cm × 0.5 cm cubes (that is, explants) and infiltrated with *Agrobacterium* cultures for 10 min. Subsequently, after 3 d of co-cultivation in the dark on the co-culture medium (MS + 2.25 mg/L 6-BA + 0.3 mg/L NAA), the explants were transferred to selective medium (MS + 2.25 mg/L 6-BA + 0.3 mg/L NAA + 400 mg/L cefotaxime + 100 mg/L kanamycin). The culture was kept at 25 °C with a 16/8 h light/dark cycle with the medium changed every 15 d. When the callus and resistant shoots emerged, the explants were transferred to germination medium (MS + 0.1 mg/L 6-BA + 0.01 mg/L NAA + 400 mg/L cefotaxime + 100 mg/L kanamycin). When the shoots grew to around 2 cm in length, the explants were transferred to rooting medium (MS + 400 mg/L cefotaxime + 100 mg/L kanamycin). Finally, after the roots were developed and mature, the plantlets were removed from the medium and planted in sterile soil.

After one month of growth, a Plant Rapid Genome Extraction kit (Tsingke, Wuhan, China) was used for a positive test of pCAMBIA1300-OfYABBY12 transgenic and wild-type (WT) tobacco leaves. Positive plants and WT plants were used for semi-quantitative validation, and transgenic lines with high levels of expression were selected for phenotype observation, scanning electron microscopy, gas chromatography–mass spectrometry (GC–MS), and RNA sequencing (RNA-Seq).

### GC–MS volatile analysis

Headspace solid-phase microextraction (HS-SPME) was used to collect VOCs released from tobacco leaves or flowers. The samples of fresh leaves or flowers were ground in liquid nitrogen, 1 g of leaf or flower powder was placed in a 10-mL SPME vial with 3 mL NaCl saturated solution, and 1 µL of 5000-fold diluted ethyl caprate was added as the internal standard. Each vial was placed at room temperature (25 ± 2 °C) for 30 min and then extracted at 55 °C for 30 min using an extraction head composed of 65 µM polydimethylsiloxane (PDMS)/divinylbenzene (DVB) fiber (Supelco Co., Bellefonte, PA, USA) for GC–MS detection. GC–MS sequencing was conducted according to a previously described protocol [30].

### Scanning electron microscopy

Fresh tobacco leaf samples were fixed in formalin–acetic acid–ethyl alcohol (FAA) at 4 °C for 48 h. The samples were then dehydrated sequentially in a gradient ethanol series, and after dehydration, the samples were vacuum-dried and sprayed with a gold film layer. Finally, the samples were placed under an environmental scanning electron microscope (Quanta 200, FEI Inc, Netherlands) for observation and photographs.

### RNA-Seq analysis

Total RNA was extracted from mature WT and *OfYABBY12*-overexpressing (*OfYABBY12-OE*) tobacco leaves using an RNAprep Pure Plant Kit (Tiangen, Beijing, China). Each sample had three biological replicates. RNA-Seq was performed by the Guangzhou Gene Denovo Biotechnology Co. (Guangzhou, China) using the Illumina HiSeq2500 platform. All sequencing data were uploaded to the National Center for Biotechnology Information (NCBI) Sequence Read Archive (SRA) database (https://www.ncbi.nlm.nih.gov/sra/) under the accession number SRP450701.

### Dual-luciferase transient expression assay

The coding sequence (CDS) of *OfYABBY12* was amplified via PCR using gene-specific primers (Table S2) and connected to the pGreenII 62-SK vector to generate an effector, which was transformed into the *A*. *tumefaciens* strain GV3101 (pSoup). Then, the *A*. *tumefaciens* cells carrying the effector and reporter (pGreenII 0800-LUC-OfCCD4, preserved by our group) plasmids were mixed in a 4:3 ratio (v/v) and injected into *N. benthamiana* leaves. After 48 h, the luciferase (LUC) signal was detected using BERTHOLD NightSHADE LB 985 (Berthold, Bad Wildbad, Germany), and LUC and renilla luciferase (REN) were detected using a Dual-Luciferase Reporter Assay System kit (Yeasen Biotechnology, Shanghai, China).

### Statistical analysis

One-way analysis of variance (ANOVA), Duncan’s multiple range test, and Student’s t-test were performed using IBM SPSS Statistics (version 20.0). A *p*-value smaller than 0.05 was considered significant. A minimum of three biological replicates were utilized for all experiments. Bar graphs were created using Origin Pro 2018. Heat maps were generated using TBtools software (version 1.120). Principal component analysis (PCA) and orthogonal partial least squares discrimination analysis (OPLS-DA) of VOCs were performed using SIMCA-13.0 software (version 13.0.3).

## Results

### Identification and classification of YABBY gene family members in *O. fragrans*

**Fig. 1:**
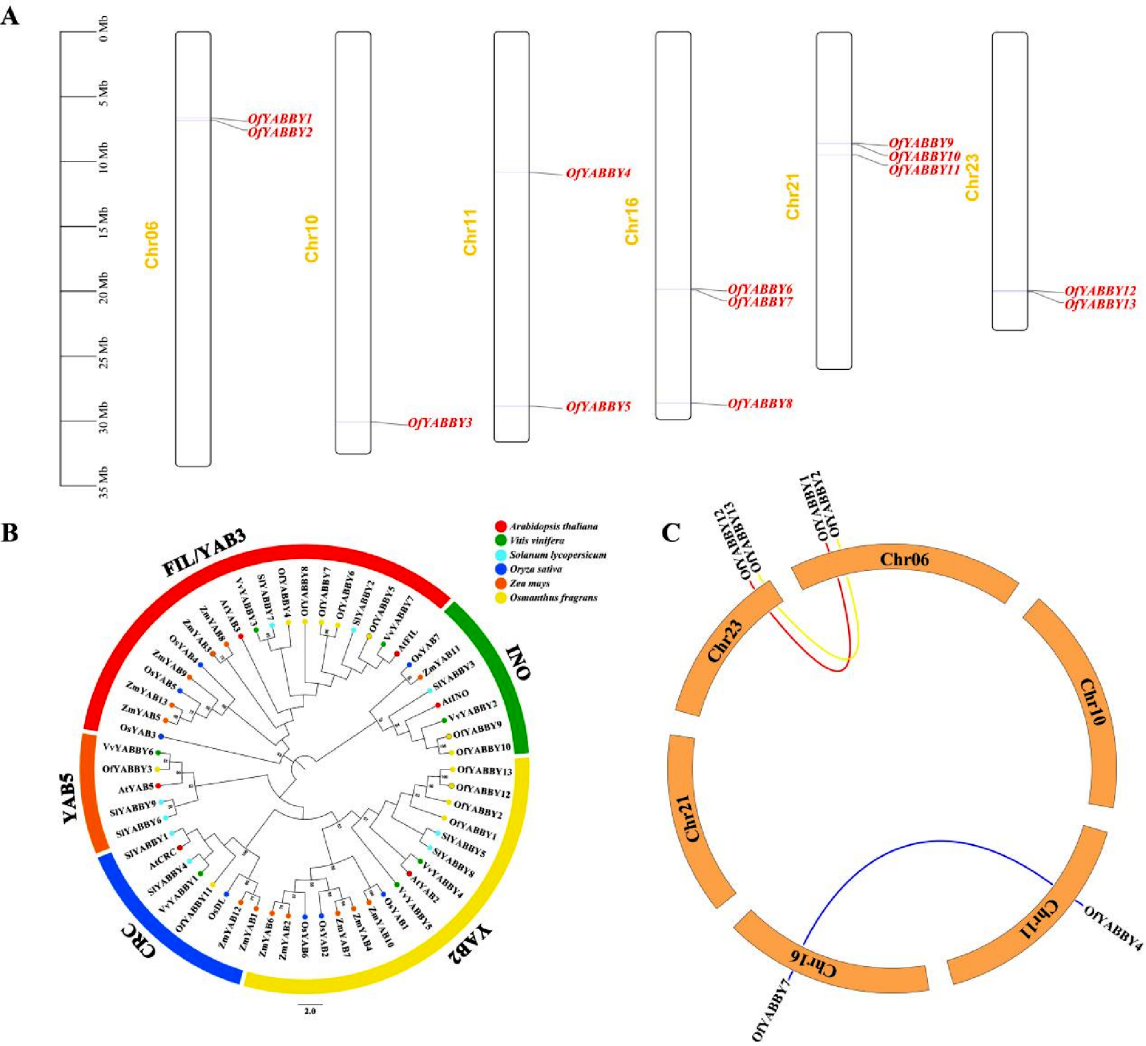
Chromosomal distribution, phylogenetic relationships, and collinearity analysis of *Osmanthus fragrans YABBY* genes. (**A**) Chromosome locations of the *OfYABBY* genes. (**B**) Phylogenetic analysis of YABBYs from *O*. *fragrans*, *Arabidopsis thaliana*, *Vitis vinifera*, *Solanum lycopersicum*, *Oryza sativa* and *Zea mays*. Numbers at the nodes indicate bootstrap values; values lower than 50% are not shown. The sequences of the YABBYs used for phylogenetic relationship analysis are listed in Table S5. (**C**) Collinearity analysis for all *OfYABBY* genes

In this study, HMMER was used to identify 19 candidate *YABBY* genes in the *O*. *fragrans* whole genome database, and six sequences without intact structural domains were removed using NCBI-CDD, Pfam, and SMART. A total of 13 *YABBY* genes were identified in the *O*. *fragrans* genome. The *OfYABBY* genes were named based on their position on chromosomes as *OfYABBY1–OfYABBY13*, and these 13 genes were located on *O*. *fragrans* chromosomes 6, 10, 11, 16, 21, and 23 (Fig. 1A). The length of OfYABBY proteins ranged from 149 aa (OfYABBY13) to 247 aa (OfYABBY2), and the protein molecular weight ranged from 16.69 kDa (OfbYABBY13) to 27.39 kDa (OfbYABBY2). The theoretical isoelectric point (pI) was 6.18–9.3, and OfYABBY5, OfYABBY8, OfYABBY9, and OfYABBY10 were acidic proteins (pI < 7.00), while the remaining proteins were basic (Table S3).

The sequence alignment results of the 13 OfYABBY proteins revealed that all OfYABBY proteins contained two highly conserved protein domains, of which one was a C_2_C_2_ zinc finger domain in the N terminal, and one was a YABBY domain in the C terminal (Fig. S1A). In addition, the 13 *OfYABBY* genes contained four to seven introns, of which most *OfYABBY* genes contained six introns. *OfYABBY5* and *OfYABBY*8 contained the greatest number of introns at seven. Overall, *OfYABBY* genes that clustered together shared identical or similar exon/intron structures. The conserved motifs of the OfYABBY family were analyzed (Table S4). All members contained at least two motifs, motifs 1 and 2, and genes that clustered together had similar motifs (Fig. S1B). These genes may have similar functions.

Interactions between cis- and trans-acting factors can regulate gene expression. The promoters and their upstream cis-acting elements were analyzed to predict their corresponding gene functions. In the *O*. *fragrans YABBY* gene family, every gene promoter contained four to 10 cis-acting elements (Fig. S2). Most were considered involved in light response, including GT1-motif, Box 4, TCT-motif, G-Box, AE-box, GA-motif, ATCT-motif, GATA-motif, chs-CMA1a, 3-AF1 binding, I-box, AE-box, TCCC-motif, MRE, and ACE. The responses to gibberellin, abscisic acid, salicylic acid, auxin, and methyl jasmonate included TATC-box, P-box, GARE-motif, ABRE, TCA-element, TGA-box, TGA-element, TGACG-motif, and CGTCA-motif. LTR, MBS, ARE, and TC-rich repeats might be involved in responding to drought, anaerobic, and low temperature stress. In addition, cis-acting elements such as CAT-box and HD-Zip 1 were involved in the developmental regulation of plant tissues and organs. This means that *OfYABBY*s may be involved in the responses to many plant hormones and environmental stress and play a role in plant growth and development.

### Phylogenetic analysis and gene duplication

To understand the phylogenetic relationship between various YABBYs, 56 YABBY proteins from dicotyledonous plants (*O*. *fragrans*, *A*. *thaliana*, *S*. *lycopersicum*, and *V*. *vinifera* [15]) and monocotyledonous plants (*O*. *sativa* and *Z*. *mays*) were used to construct a phylogenetic tree (Fig. 1B). Based on the classification of *A. thaliana* YABBY proteins [39], the 13 OfYABBYs were divided into five subfamilies, namely FIL/YAB3, YAB5, YAB2, CRC, and INO. The FIL/YAB3 subfamily included five OfYABBY proteins, and the YAB2 subfamily included four OfYABBY proteins. In addition, the INO subfamily included two YABBY proteins, and the CRC and YAB5 subfamilies each included one OfYABBY (Table S3). As predicted, phylogenetic analysis showed that OfYABBYs had the closest phylogenetic relationship to YABBYs from dicotyledonous plants. The YABBYs of dicotyledonous plants were all clustered together. Moreover, the YABBYs of monocotyledonous plants, such as rice (*O*. *sativa*), formed a parallel branch, consistent with previous reports [9]. This shows that *YABBY* genes may function in the differentiation of monocotyledonous and dicotyledonous plants and that they are conserved in dicotyledonous plants.

In addition, phylogenetic analysis of *O*. *fragrans* YABBY protein sequences was conducted based on YABBYs in *M*. *lewisii*, *C*. *sinensis*, *C*. *praecox*, *A*. *annua*, and *M*. *spicata*. This is because their functions have been sufficiently discussed, such as *MlYAB1*/*2*/*3*/*5*, *CsFILa*/*b*, and *CsYAB2*, which are involved in leaf development regulation [33, 34], and *CpFIL*, *CpCRC*, *CpYABBY2*, *CpYABBY5-1*/*-2*, *AaYABBY5* and *MsYABBY5*, which are involved in secondary metabolite biosynthesis [8, 17, 18, 35]. Based on the results (Fig. S3), it was deduced that the functions of *OfYABBY1*/*2*/*3*/*4*/*5*/*6*/*7*/*8*/*12*/*13* genes of the FIL/YAB3, YAB2, and YAB5 subfamilies in *O. fragrans* may be related to leaf development or secondary metabolite synthesis. In addition, as shown in Fig. 1B, the FIL/YAB3 and YAB2 subfamilies contained more *OfYABBY* genes than other subfamilies, exhibiting significant gene duplication. However, because the number of *O*. *fragrans* YABBY family members was low, gene collinearity analysis did not find tandem repeat *OfYABBY* genes, but three segmental duplication of *OfYABBY* genes (*OfYABBY1/OfYABBY12*, *OfYABBY2/OfYABBY13*, and *OfYABBY4/OfYABBY7*) were detected (Fig. 1C).

### Expression patterns of OfYABBYs

**Fig. 2:**
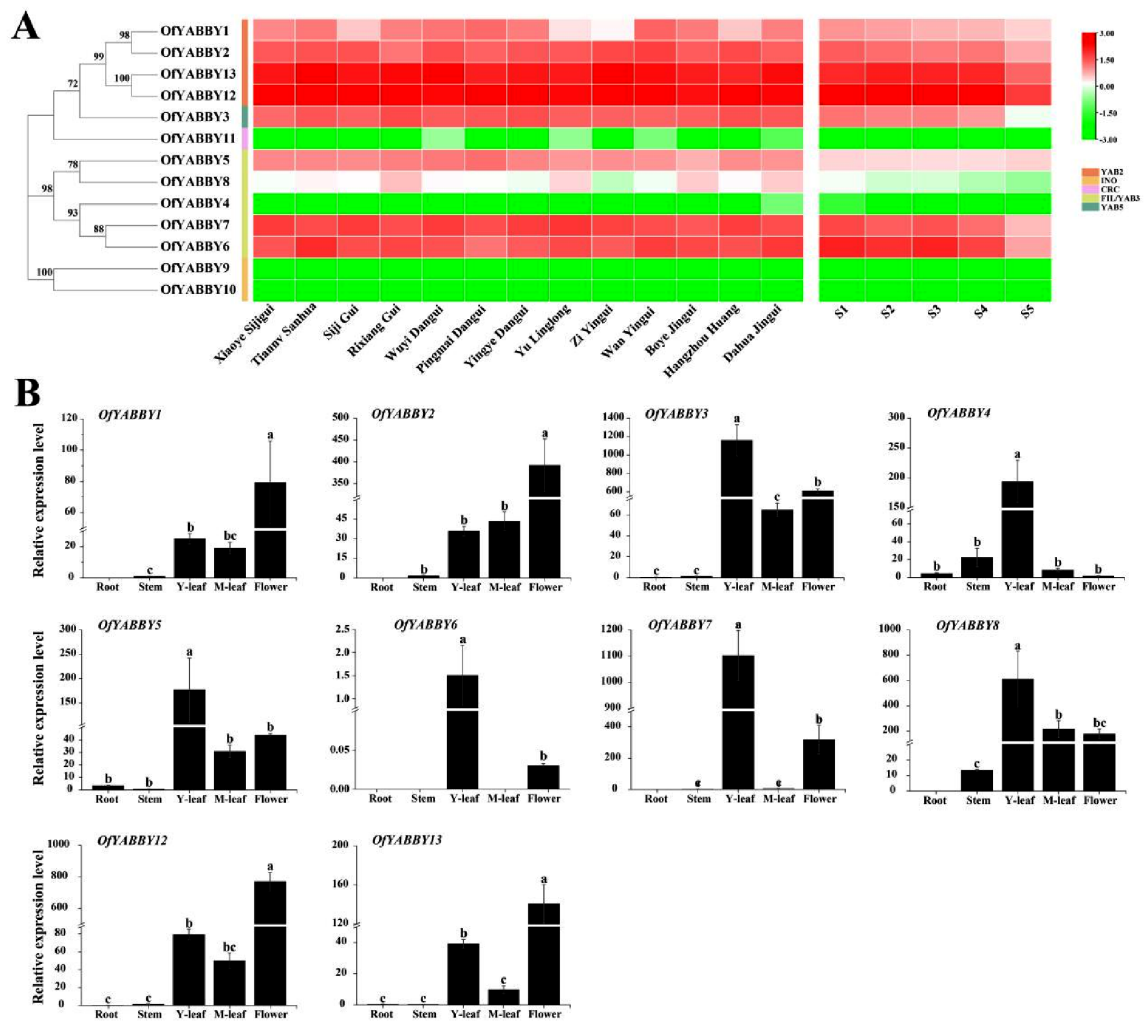
Expression analysis of *OfYABBY* genes. (**A**) Differential expression profiles of *OfYABBY* genes in 13 cultivars at the full blooming stage, and five flowering stages including the bud-pedicel stage (S1), bud-eye stage (S2), primary blooming stage (S3), full blooming stage (S4), and flower fading stage (S5) of *Osmanthus fragrans* ‘Rixiang Gui’ based on RNA sequencing data. (**B**) Expression patterns of *OfYABBY* genes were confirmed in roots, stems, leaves (young and mature leaves), and flowers (full blooming stage) using quantitative reverse-transcription polymerase chain reaction (qRT-PCR) analysis. Error bars indicate the standard deviations of three biological replicates. Different letters indicate a significant difference (*p* < 0.05) as determined by analysis of variance (ANOVA), which is based on Duncan’s multiple range test

Transcriptome data were obtained to analyze the expression patterns of *OfYABBY*s in different *O*. *fragrans* cultivars [40] and different flowering stages (bud-pedicel stage, bud-eye stage, primary blooming stage, full blooming stage, and flower fading stage) (unpublished, Table S6). The results showed that two *OfYABBY* genes in the INO subfamily (*OfYABBY9* and *OfYABBY10*) and *OfYABBY11* in the CRC subfamily had limited expression or were not expressed in floral tissues. Previous studies have reported that INO and CRC subfamily genes are only expressed in reproductive organs, and it is hypothesized in this study that *OfYABBY*s in the CRC and INO subfamilies may not participate in the formation of floral organs. Unlike *OfYABBY9*, *OfYABBY10*, and *OfYABBY11*, *OfYABBY* genes in the FIL/YAB3, YAB2, and YAB5 subfamilies, except from *OfYABBY4* and *OfYABBY8*, were highly expressed in floral tissues, especially *OfYABBY12* and *OfYABBY13* (Fig. 2A).

This study further conducted qRT-PCR analysis of the expression levels of 10 *OfYABBY* genes from the FIL/YAB3, YAB2, and YAB5 subfamilies in the roots, stems, leaves (young and mature leaves), and floral tissues. The expression patterns of various *OfYABBY*s in different tissues showed significant differences (Fig. 2B). Overall, these 10 *OfYABBY* genes were slightly or not expressed in roots and stems. Among them, *OfYABBY4/5/6/7/8* from the FIL/YAB3 subfamily exhibited preferential expression in young leaves, and *OfYABBY3* from the YAB5 subfamily had low expression in roots, stems, and mature leaves, and highest expression in young leaves, followed by flowers. Notably, *OfYABBY1*/2*/12/13* exhibited similar expression patterns, with significantly higher expression in flowers than in other tissues.

### Subcellular localization and transcriptional activation activity of OfYABBYs

**Fig. 3:**
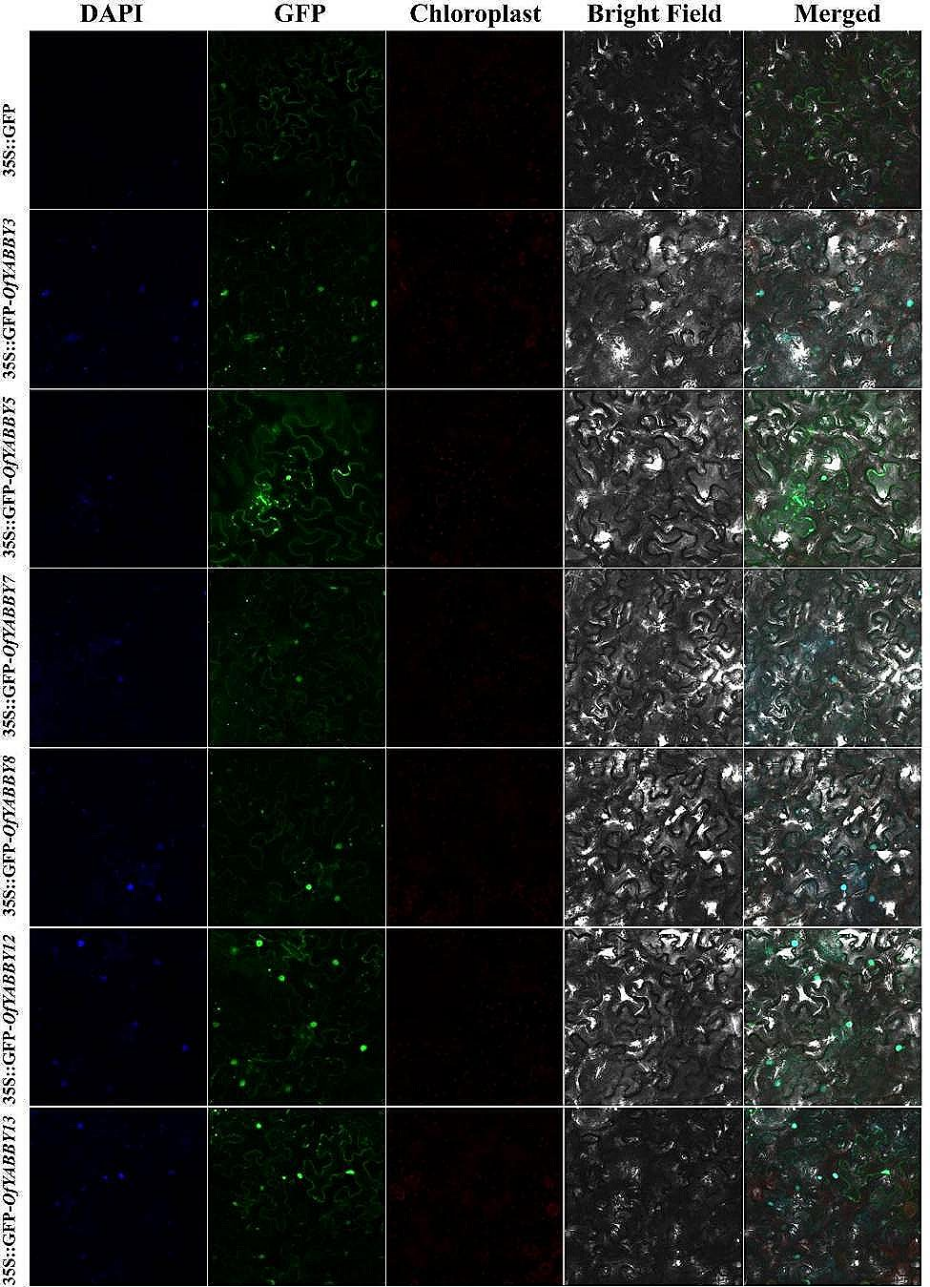
Subcellular localization of selected OfYABBY proteins. The OfYABBY proteins fused with green fluorescent protein (GFP) were transiently expressed in tobacco leaf cells to observe subcellular localization through laser confocal microscopy. GFP fluorescence is shown in green and the nucleus is blue with 4’,6-diamidino-2-phenylindole (DAPI) staining

**Fig. 4:**
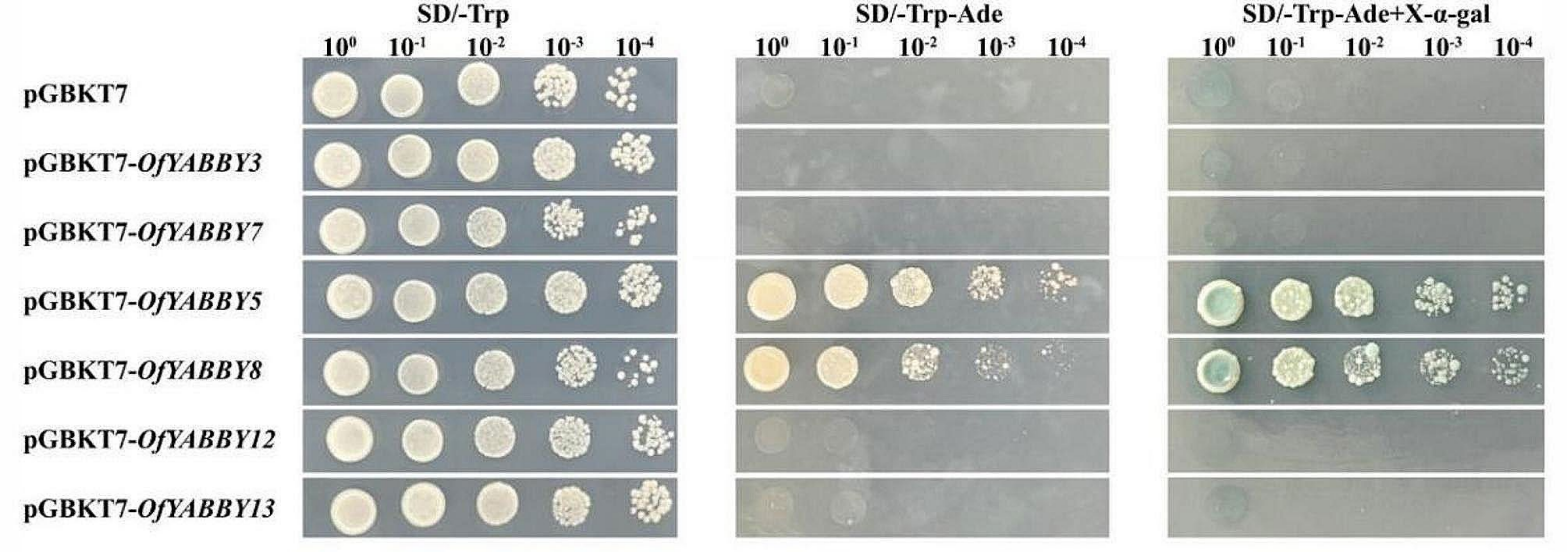
Transcriptional activation analysis of selected OfYABBY proteins. The transformed yeast cells containing pGBKT7 and pGBKT7-OfYABBYs were spread on SD/-Trp, SD/-Trp-Ade, and SD/-Trp-Ade + X-α-gal media

To examine the potential function of *OfYABBY* genes in transcriptional regulation, this study selected six *OfYABBY* genes from the FIL/YAB3, YAB2, and YAB5 subfamilies for subcellular localization analysis based on the bioinformatics analysis. Laser confocal microscopy revealed that the green fluorescent protein (GFP) fluorescence signals of 35 S::GFP-OfYABBY3/5/7/8/12/13 fusion proteins were detected mainly in the cell nuclei (Fig. 3). In addition, this work further cloned these six *OfYABBY* genes into the yeast expression vector pGBKT7 and found that yeast strains carrying the pGBKT7 empty vector (negative control) and the pGBKT7-OfYABBY vectors exhibited good growth on SD/-Trp culture medium. Only pGBKT7-OfYABBY5/8-transformed yeast could grow on SD/-Trp-Ade culture medium, and these strains showed normal growth and blue colonies on X-α-gal containing SD/-Trp-Ade culture medium (Fig. 4). This indicates that OfYABBY5/8 shows transcriptional activity in yeast, while OfYABBY3/7/12/13 does not show transcriptional activity.

### Phenotypes of transgenic *N. tabacum* overexpressing OfYABBY12

Plant YABBY transcription factors participate in the development of plant leaves and floral organs, as well as the biosynthesis of secondary metabolites, such as floral VOCs. Therefore, this study selected *OfYABBY12*, significantly more highly expressed in *O. fragrans* flowers than in other organs, for heterologous overexpression in tobacco. This allowed the further examination of the potential function of *OfYABBY*s. Positive transgenic tobacco plants were selected using semi-quantitative RT-PCR, and 10 transgenic lines were obtained (Fig. S4). Three lines with higher expression levels (lines 2, 5, and 12) were selected for subsequent analysis.

**Fig. 5:**
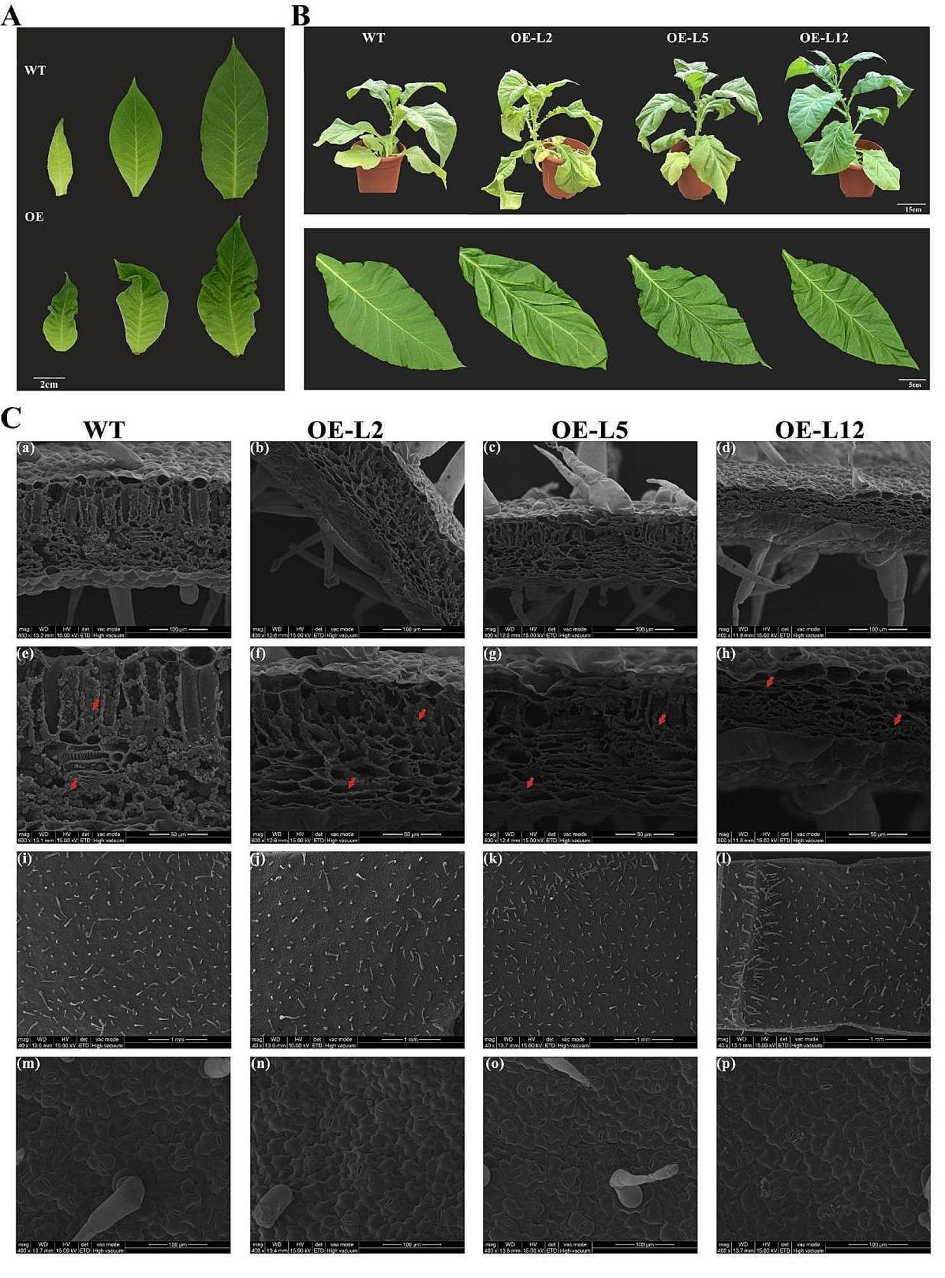
Phenotypic analysis of *OfYABBY12* transgenic plants. (**A**) Leaf morphology of the wild-type (WT) and *OfYABBY12-OE* lines (overexpression of *OfYABBY12* in tobacco (OE)) at different developmental stages. (**B**) *OfYABBY12* transgenic plants produced curled leaves when compared with the WT. (**C**) Scanning electron microscopic images of tobacco leaves. (a–h) Transverse sections of tobacco leaves. (i–p) Epidermal structures of tobacco leaves. Red arrows show the inclusions in the tobacco leaves

The phenotypic observation of tobacco leaves showed that, compared with the WT, the most intuitive phenotypic difference was that wrinkles appeared at the early stages of development in *OfYABBY12-OE* tobacco leaves and became more severe (Fig. 5A). Compared with WT tobacco, leaves in the overexpressing lines exhibited significant downward curvature and wrinkling (Fig. 5B), indicating that *OfYABBY12* participated in the establishment of abaxial polarity in tobacco leaves. Scanning electron microscopy images of tobacco leaves (Fig. 5C) showed that the cross-sections of leaves from overexpression lines were significantly thinner, while the palisade and spongy mesophyll tissues were denser compared with WT tobacco (panels a–d). The leaf thickness of OfYABBY12-OE in lines 2, 5, and 12 decreased by 33, 33, and 57%, respectively, compared with the WT (Fig. S6). It was also observed that WT tobacco leaves were filled with granular inclusions, while there were significantly fewer inclusions in overexpressing lines (panels e–h). However, no significant difference was observed in leaf epidermal cells. Specifically, no significant differences were observed in epidermal hair (panels i–l) or stomata (panels m–p).

Phenotypic observation of tobacco flowers showed that the pistil (style) of overexpressing tobacco lines was significantly extended compared with the WT, while the stamen length remained unchanged after *OfYABBY12* gene expression was upregulated (Fig. S7).

### Detection of VOCs in transgenic N. tabacum overexpressing OfYABBY12

**Fig. 6:**
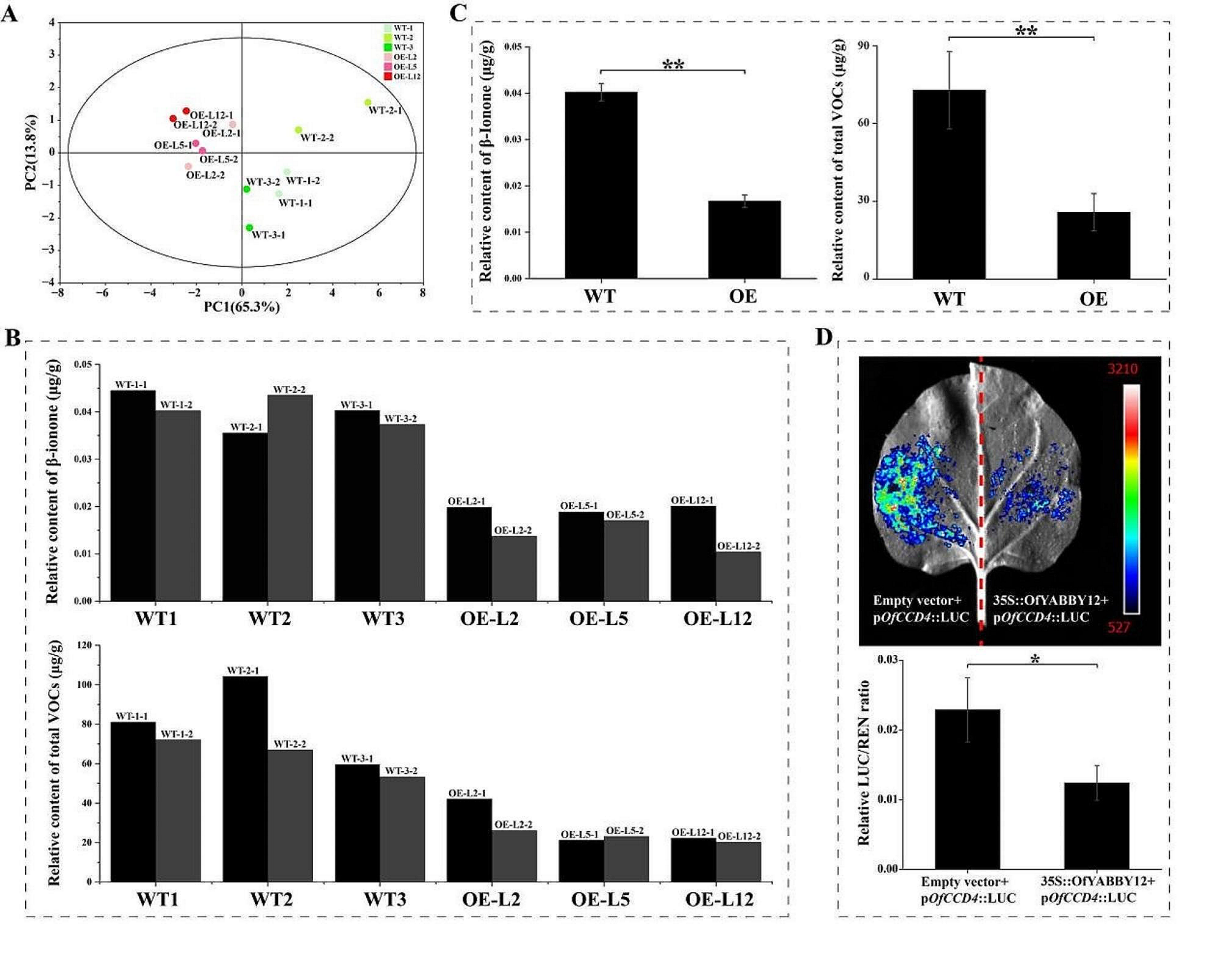
Effects of *OfYABBY12* overexpression on volatile organic compound (VOC) metabolism and the structure of tobacco leaves. (**A**) Principal component analysis (PCA) of VOCs in wild-type (WT) and *OfYABBY12*-overexpressing (*OfYABBY12-OE*) leaves. (**B**, **C**) Relative content analysis of β-ionone and total compounds of tobacco leaves. Two replicates per strain are shown in (**B**), and the means calculated from three strains with error bars reflecting standard deviations are shown in (**C**). (**D**) The dual-luciferase assay verified the relationship between OfYABBY12 and Pro-*OfCCD4*. Renilla (REN) luminescence was used to normalize the luciferase (LUC) activity. The mean ± standard deviation (SD) from three replicates is shown. Asterisks indicate significant differences (Student’s t-test: *P < 0.05; **P < 0.01)

To further determine the function of *OfYABBY12*, this study assessed VOC levels in the leaves of *OfYABBY12-OE* tobacco plants. PCA revealed significant metabolic differences between WT and *OfYABBY12-OE* tobacco leaves (Fig. 6A), and variable importance in projection (VIP) values from OPLS-DA showed that β-ionone was the key differential metabolite between WT and *OfYABBY12-OE* tobacco leaves (Table S7). In *OfYABBY12-OE* tobacco leaves, the β-ionone content and total VOC content were both significantly lower than in the WT leaves (Figs. 6B and C). However, the GC–MS of the tobacco flowers showed no significant differences in VOCs released from WT and *OfYABBY12-OE* tobacco, and β-ionone was not detected in any of the tobacco flowers. PCA also showed that VOCs released by WT and overexpressing tobacco flowers could not be differentiated (Fig. S8).

In *O*. *fragrans*, carotenoid cleavage dioxygenase gene 4 (*CCD4*) positively regulates β-ionone biosynthesis [41, 42]. Thus, this study verified the regulatory relationship between OfYABBY12 and the *OfCCD4* promoter using a dual-luciferase assay. Tobacco leaf cells had strong LUC signals, but the LUC/REN ratio of the experimental group (35 S::OfYABBY12 + p*OfCCD4*::LUC) was significantly lower than that of the control group (empty vector + p*OfCCD4*::LUC), indicating that OfYABBY12 negatively regulated the expression of *OfCCD4* (Fig. 6D).

**Fig. 7:**
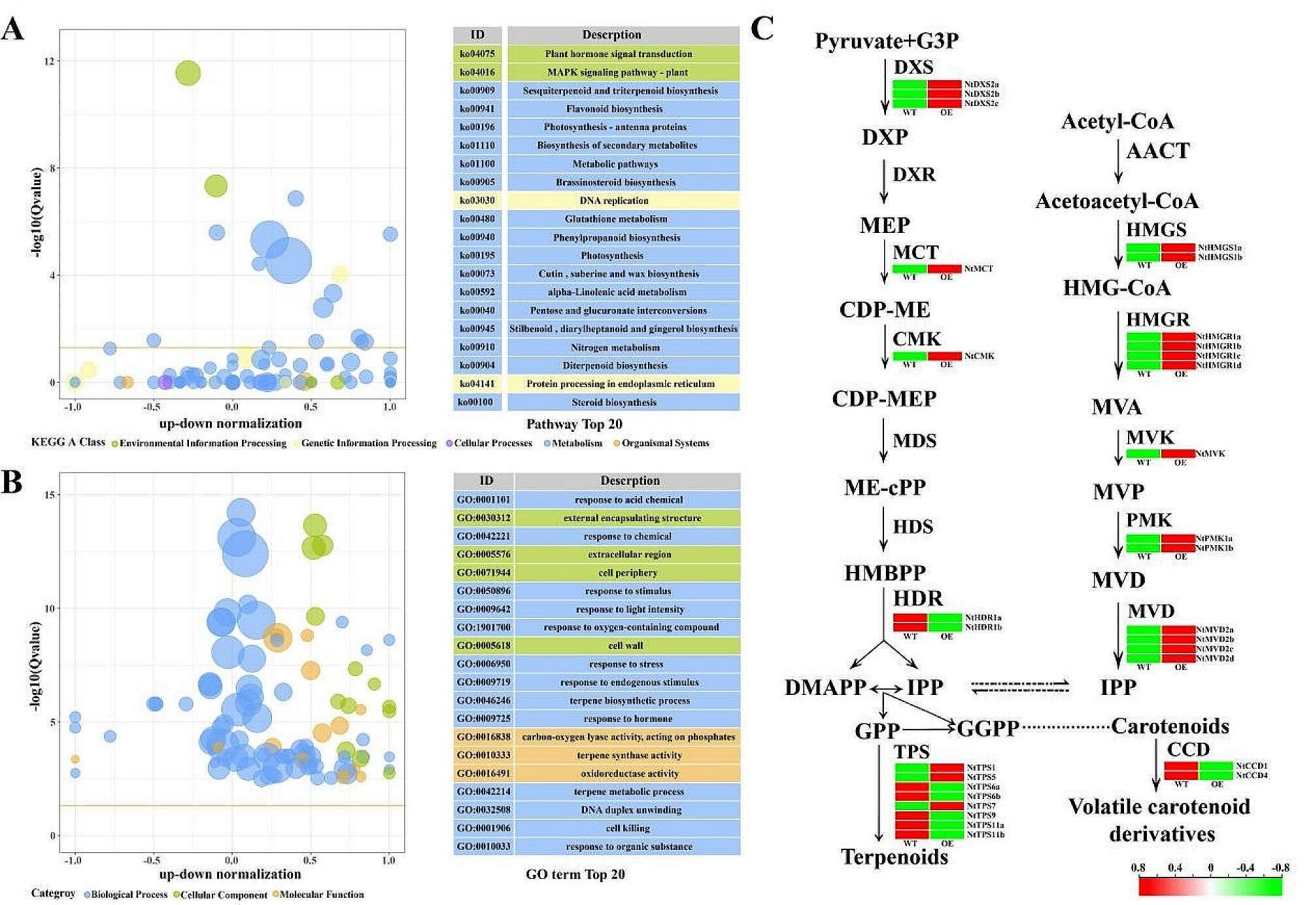
Transcriptome profiling of tobacco leaves. (**A**) Kyoto Encyclopedia of Genes and Genomes (KEGG) pathway analysis of differentially expressed genes (DEGs) in transgenic leaves. (**B**) Gene Ontology (GO) classification of unigenes among the annotated DEGs of *OfYABBY12* overexpression in transgenic leaves. The bubble chart shows the enrichment of DEGs in certain pathways. (**C**) Overview of transcript changes in terpene biosynthesis pathway genes in wild-type (WT) and *OfYABBY12*-overexpressing (*OfYABBY12-OE*) leaves. DXS: 1-deoxy-D-xylulose-5-phosphate synthase; DXR: 1-deoxy-D-xylulose-5-phosphate reductoisomerase; MCT: 2-C-methyl-D-erythritol 4-phosphate cytidylyltransferase; CMK: 4-diphosphocytidyl-2-C-methyl-D-erythritol kinase; MDS: 2-C-methyl-D-erythritol 2,4-cyclodiphosphate synthase; HDS: (E)-4-hydroxy-3-methylbut-2-enyl-diphosphate synthase; HDR: 4-hydroxy-3-methylbut-2-enyl diphosphate reductase; AACT: acetyl-CoA C-acetyltransferase; HMGS: 3-hydroxy-3-methylglutaryl-CoA synthase; HMGR: 3-hydroxy-3-methylglutaryl CoA reductase; MVK: mevalonate kinase; PMK: phosphomevalonate kinase; MVD: diphosphomevalonate decarboxylase; TPS: terpene synthase; CCD: carotenoid cleavage dioxygenase

Subsequently, RNA-Seq analysis was conducted on tobacco leaves to study the effects of *OfYABBY12* overexpression on the transcription levels in overexpressing tobacco. A total of 3721 differentially expressed genes (DEGs, expression change > 2-fold, *p* < 0.05) were identified (Fig. S9). The Kyoto Encyclopedia of Genes and Genomes (KEGG) analysis of DEGs showed that these DEGs mainly participate in signaling pathways and metabolism in plants. Most DEGs found in the present study were concentrated in secondary metabolite synthetic pathways, such as in the biosynthesis of terpenes, flavonoids, and phenylpropanoids (Fig. 7A). In addition, Gene Ontology (GO) enrichment analysis of these DEGs found three GO entries in the top 20 enriched items related to terpene biosynthesis (Fig. 7B). Analysis of the DEGs that participate in terpene biosynthesis found that most upstream genes in the metabolic pathway were upregulated in the overexpressing lines. Among downstream genes, some *NtTPSs* were downregulated, and some were upregulated. *NtCCD1* and *NtCCD4* were significantly downregulated (Fig. 7C).

## Discussion

### Overview of the YABBY gene family in *O. fragrans*

The *YABBY* gene family has relatively low membership in different species and is a small gene family. For example, six, nine, and seven *YABBY* gene members have been identified in *A*. *thaliana* [39], *S*. *lycopersicum* [43], and *V*. *vinifera* [15], respectively. In this study, 13 *OfYABBY* genes were identified in the *O*. *fragrans* genome. This relatively high number may be the result of gene duplication events. This study did not find tandem repeats of the *YABBY* genes in *O*. *fragrans*. However, three segmental duplication *YABBY* genes were identified (Fig. 1C), indicating that the evolution of the *O*. *fragrans YABBY* gene family may have been driven by segmental duplication. In addition, genomic studies have shown that *O*. *fragrans* underwent two whole genome duplication events compared with *A*. *thaliana* and *V*. *vinifera* [30].

*OfYABBY*s in *O*. *fragrans* could be divided into five subfamilies based on phylogenetic and gene structural analysis. Genes in the same subfamily showed similar motifs and intron/exon structures, suggesting that they may have similar functions. In addition, an analysis of the promoter regions of *O*. *fragrans YABBY* genes showed that photo-responsive elements were present in all members and were the cis-acting elements with the highest quantity. These genes may play an important role in photo-response and photomorphogenesis, thereby affecting the growth and development of plant leaves. Moreover, some cis-acting elements involved in the developmental regulation of plant tissues and organs were identified. Previous studies have also reported that YABBY gene family members play extremely important roles in leaf development and synthesis [44–47]. In addition, the cis-acting elements include some plant hormone response elements, such as abscisic acid, auxin, and methyl jasmonate. One study reported that the interactions between *A*. *thaliana* FIL and JAZ proteins (an inhibitor of the jasmonic acid pathway) affected anthocyanidin accumulation [48]. *O. fragrans OfYABBY1/2/4/6/7/8/9/10/11/12/13* all contain methyl jasmonate response elements. We speculate that these *OfYABBY* genes may be mediated by jasmonic acid signaling to regulate the synthesis of various secondary metabolites.

The function of *O*. *fragrans YABBY* genes can be predicted to some extent based phylogenetic relationships with previously studied *YABBY* genes. Consistent with previous studies, the *O*. *fragrans* YABBY genes were found to have closer phylogenetic relationships with YABBYs from dicotyledon plants than with those from monocotyledon plants (*A*. *thaliana*, *S*. *lycopersicum*, and *V*. *vinifera*) (Fig. 1B). As important model plants, the functions of the *A*. *thaliana* and *S*. *lycopersicum YABBY* genes have been extensively examined. These genes mainly play important roles in plant growth and development, particularly in leaf growth, fruit development, floral organ formation, and the synthesis of plant secondary metabolites [14, 27, 49, 50]. In addition, subcellular localization analysis showed that 35 S::GFP-OfYABBY3/5/7/8/12/13 fusion proteins were mainly located in the nucleus, suggesting that these proteins have diverse functions in the nucleus. Transcriptional activity analysis revealed that OfYABBY5/8 exhibited transcriptional activity in yeast cells and may directly regulate downstream target genes. However, OfYABBY3/7/12/13 did not exhibit transcriptional activity in yeast cells, and thus, these proteins may require interactions with other transcription factors or specific environmental conditions to perform their regulatory functions.

### OfYABBY12 plays a negative role in VOC synthesis

YABBY transcription factors have been reported to have tissue specificity in plants and to play different roles in different plant tissues [44]. In the present study, *OfYABBY9*, *OfYABBY10*, and *OfYABBY11* were not detected or exhibited extremely low transcriptional expression in different cultivars or different flowering stages in *O*. *fragrans* floral organs. In *A*. *thaliana*, *AtINO* and *AtCRC* are only expressed in reproductive organs [22, 23]; *OfYABBY9*, *OfYABBY10*, and *AtINO* are in the same subfamily, and *OfYABBY11* and *AtCRC* in the same subfamily (Fig. 1B). This suggests that *O. fragrans* CRC and INO subfamily genes may not participate in regulating the development of floral organs. However, the FIL/YAB3, YAB2, and YAB5 subfamily genes, including *OfYABBY1/2/3/6/7/12/13*, are highly expressed in *O*. *fragrans* leaves or flowers but lowly expressed or not expressed in roots and stems. This suggests that these genes may play important roles in *O*. *fragrans* leaves and floral organs. Furthermore, studies have reported that there is redundancy in the functions of genes in the FIL/YAB3, YAB2, and YAB5 subfamilies [22, 51], and these genes often participate in regulating leaf morphogenesis, the development and differentiation of lateral organs, and the biosynthesis of secondary metabolites [8, 11, 13, 17, 28].

Notably, comparing the transcript levels of all *OfYABBY*s revealed that *OfYABBY12* in the YAB2 subfamily had the highest transcript level (Fig. 2A) and showed significant differences in expression levels among *O*. *fragrans* roots, stems, young leaves, mature leaves, and floral organs (Fig. 2B). Therefore, this study selected *OfYABBY12* for further functional validation. However, a stable genetic transformation system for *O*. *fragrans* has not yet been established. Thus, *OfYABBY12* was stably overexpressed in tobacco to determine whether OfYABBY12 regulates the differentiation and formation of tobacco leaves and floral organs, as well as whether it affects their volatile metabolites. In the present study, RNA-Seq analysis of WT and *OfYABBY12-OE* tobacco leaves found that most DEGs were enriched in metabolic pathways related to the synthesis of terpenes, flavonoids, and phenylpropanoids (Figs. 7A and B). GC–MS results showed that there were significant differences in the VOCs between WT and overexpressing tobacco leaves. The content of β-ionone and total VOCs were significantly decreased (Figs. 6A and C), and critical genes that participate in β-ionone synthesis, such as *NtCCD1* and *NtCCD4*, were significantly downregulated in overexpressing tobacco leaves (Fig. 7C). It has also been reported that *MsYABBY5* negatively regulates the synthesis of volatile terpene compounds in *M*. *spicata* [8]. β-Ionone is a critical compound for floral scent synthesis in *O*. *fragrans* [52]. Our previous study also found that *OfYABBY12* in the molecular regulatory network of β-ionone synthesis showed a significant negative correlation with critical enzyme genes in its metabolic pathway (Fig. S10) [40]. Correspondingly, the dual-luciferase assay in the present study showed that OfYABBY12 negatively regulated the expression of *OfCCD4*, which has been reported to promote β-ionone synthesis [41, 42]. The results suggest that *OfYABBY12* negatively regulates β-ionone synthesis in *O*. *fragrans*.

In addition, it was observed that the inclusions of *OfYABBY12-OE* tobacco leaves were significantly decreased compared with the WT under scanning electron microscopy (Fig. 5C), but whether this is related to the reduction of VOCs needs further study. However, *OfYABBY12* overexpression did not significantly affect VOCs from tobacco flowers. This suggests that *OfYABBY12* overexpression affects VOCs from tobacco leaves, but not from tobacco flowers. Further studies are also needed to determine the regulatory relationship.

### OfYABBY12 is involved in the development of leaves and flowers

In the present study, the overexpression of *OfYABBY12* in tobacco revealed that *OfYABBY12* overexpression affected the formation of the adaxial–abaxial axis in tobacco leaves, causing leaves to curl and crenations to occur. In *Saccharum spontaneum*, *SsYABBY2* overexpression causes the abaxial side of *A*. *thaliana* leaves to curl [53]. Overexpression of *C*. *sinensis CsFIL* and *CsYAB2* in *A*. *thaliana* causes leaves to curl [33]. Furthermore, one study found that the overexpression of the *V*. *vinifera VvYABBY4* gene in *S*. *lycopersicum* caused the pistils (stigma) of transgenic *S*. *lycopersicum* to become longer [15], while the downregulation of *MlYAB1*, *MlYAB2*, *MlYAB3*, and *MlYAB5* in *M*. *lewisii* inhibited style elongation [34]. In the present study, *OfYABBY12*, *VvYABBY4*, and *MlYAB2* were in the same subfamily (Figs. 1B and S3). Significant elongation was also observed in the pistils (stigma) of *OfYABBY12-OE* tobacco flowers, while anther length remained unchanged.

## Conclusion

The present study identified 13 *OfYABBY* genes in the *O*. *fragrans* genome, which were classified into five subfamilies. Phylogenetic analysis and gene duplication events demonstrated that gene duplication aided in the expansion of the *O*. *fragrans OfYABBY* gene family and that the gene functions of the OfYABBY family may be conserved. The genes in the YAB2, FIL/YAB3, and YAB5 subfamilies may have overlapping functions. In addition, the *OfYABBY* gene expression pattern analysis indicated that *OfYABBY* genes in the YAB2, FIL/YAB3, and YAB5 subfamilies may play important roles in *O*. *fragrans* leaf and/or floral organs. Functional validation showed that *OfYABBY12* significantly affected the VOCs from tobacco leaves, especially β-ionone, and the dual-luciferase assay revealed that OfYABBY12 negatively regulated the expression of *OfCCD4*, which promoted β-ionone synthesis. *OfYABBY12* also played an important role in the establishment of polarity in tobacco leaves and in the development of lateral organs (pistils). The above results suggest that the *OfYABBY* gene family may participate in the growth and development of *O*. *fragrans* leaves and lateral organs, as well as in the synthesis of *O*. *fragrans* floral scent. In conclusion, this study provides a foundation for further research on the *YABBY* gene family in *O*. *fragrans* as well as new findings regarding the biosynthesis of the floral scent substance β-ionone in *O*. *fragrans*.

### Electronic supplementary material

Below is the link to the electronic supplementary material.

Supplementary Material 1

Supplementary Material 2

## Data Availability

All data generated or analyzed during this study are included in this published article, its supplementary information files and publicly available repositories. RNA-Seq raw data were uploaded to the NCBI sequence read archive (http://www.ncbi.nlm.nih.gov/sra/) under accession number SRP450701 and are accessible under Bioproject archive number PRJNA997126 (http://www.ncbi.nlm.nih.gov/bioproject/).
