# Supplementary material for: Characterization of YABBY transcription factors in Osmanthus fragrans and functional analysis of OfYABBY12 in floral scent formation and leaf morphology
Source: BMC Plant Biol. 2024 Jun 21;24:589. doi: 10.1186/s12870-024-05047-y (PMC11191298; doi:10.1186/s12870-024-05047-y)
Supplement: Supplementary file 1 — Supplementary Material 1 [file 12870_2024_5047_MOESM1_ESM.docx]

| 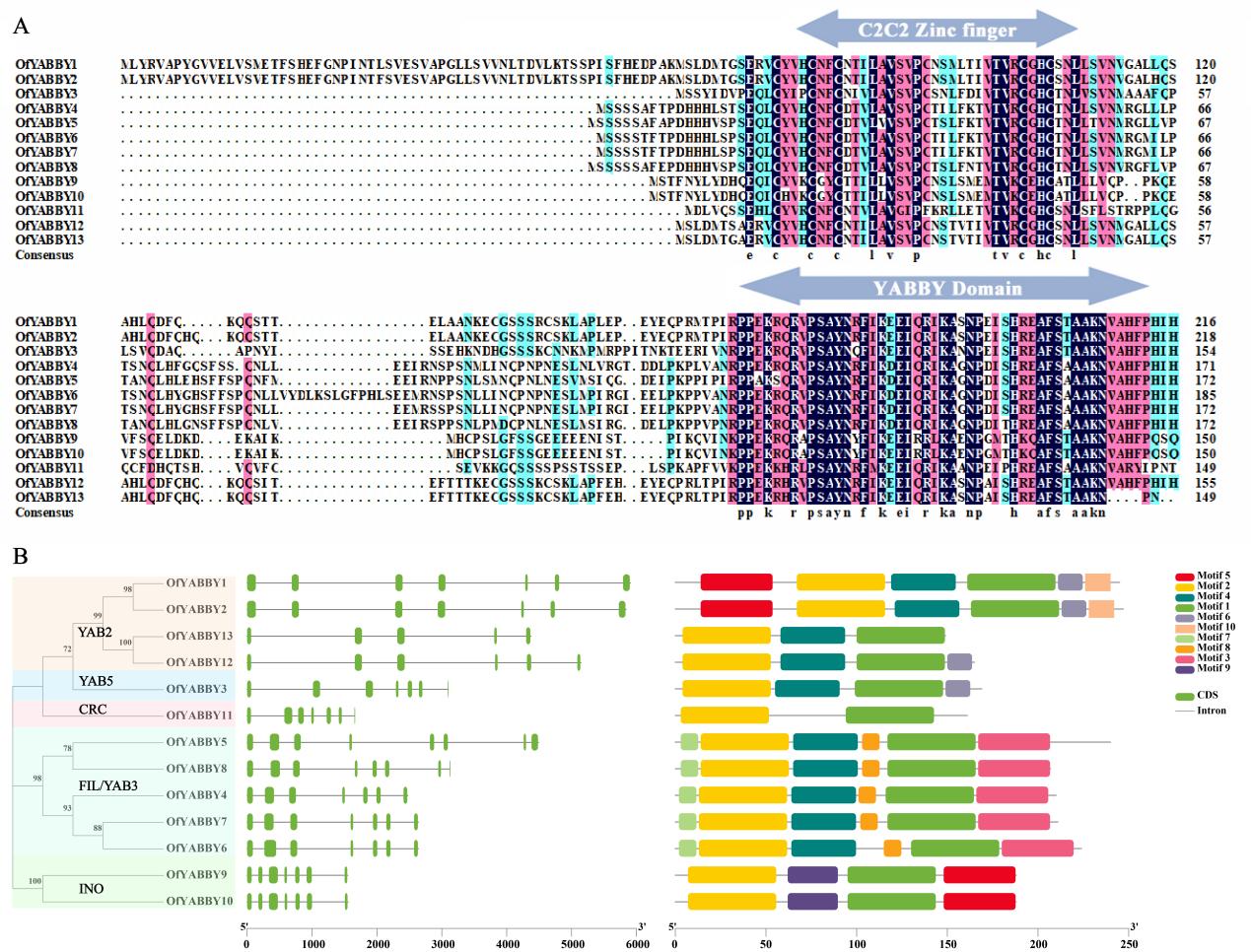 |
| --- |
| **Fig. S1** Conserved domains of the *YABBY* gene family in *Osmanthus fragrans*. (A) Members of the *YABBY* gene family are characterized by two highly conserved domains: a C2C2 zinc finger domain in the N-terminal and a YABBY domain in the C-terminal. (B) Gene structure and conserved motif analysis of 13 *OfYABBY* genes arranged according to their phylogenetic relationships. Green boxes and black lines represent exons and introns, respectively. The MEME tool was used to predict motifs; 10 motifs are shown in different colors. Detailed information on the 10 motifs is provided in Table S2. |

| 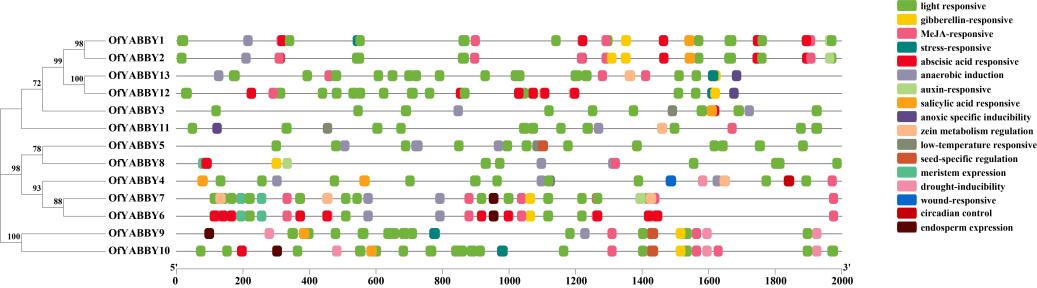 |
| --- |
| **Fig. S2** Cis-acting elements within the promoters of *OfYABBY* genes. |

| 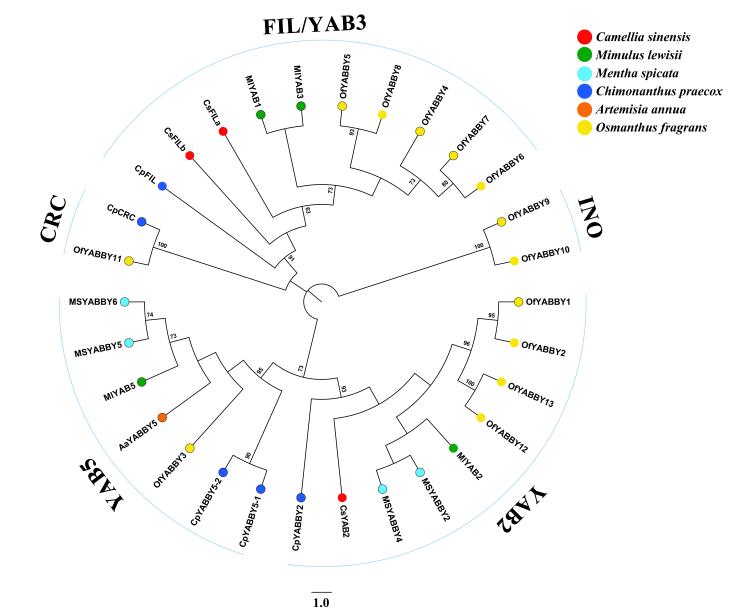 |
| --- |
| **Fig. S3** Phylogenetic analysis of YABBYs from *Osmanthus fragrans*, *Camellia sinensis*, *Mimulus lewisii*, *Mentha spicata*, *Chimonanthus praecox*, and *Artemisia annua*. The sequences of the YABBYs used for phylogenetic relationship analysis are listed in Table S5. |

| 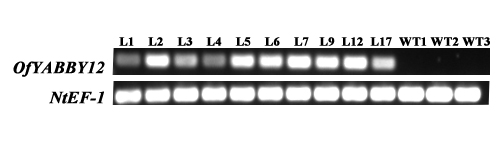 |
| --- |
| **Fig. S4** Semiquantitative RT-PCR used to determine the expression intensity of *OfYABBY12*. A fragment of the *NtEF-1* gene was amplified as the normalizer. |

| 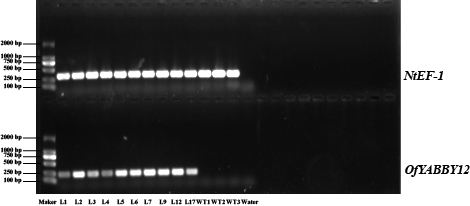 |
| --- |
| **Fig. S5** Original image for Fig. S4. Uncropped agarose gel electrophoresis results of the *NtEF-1* gene and *OfYABBY12* gene in WT and *OfYABBY12-OE* lines. |

| 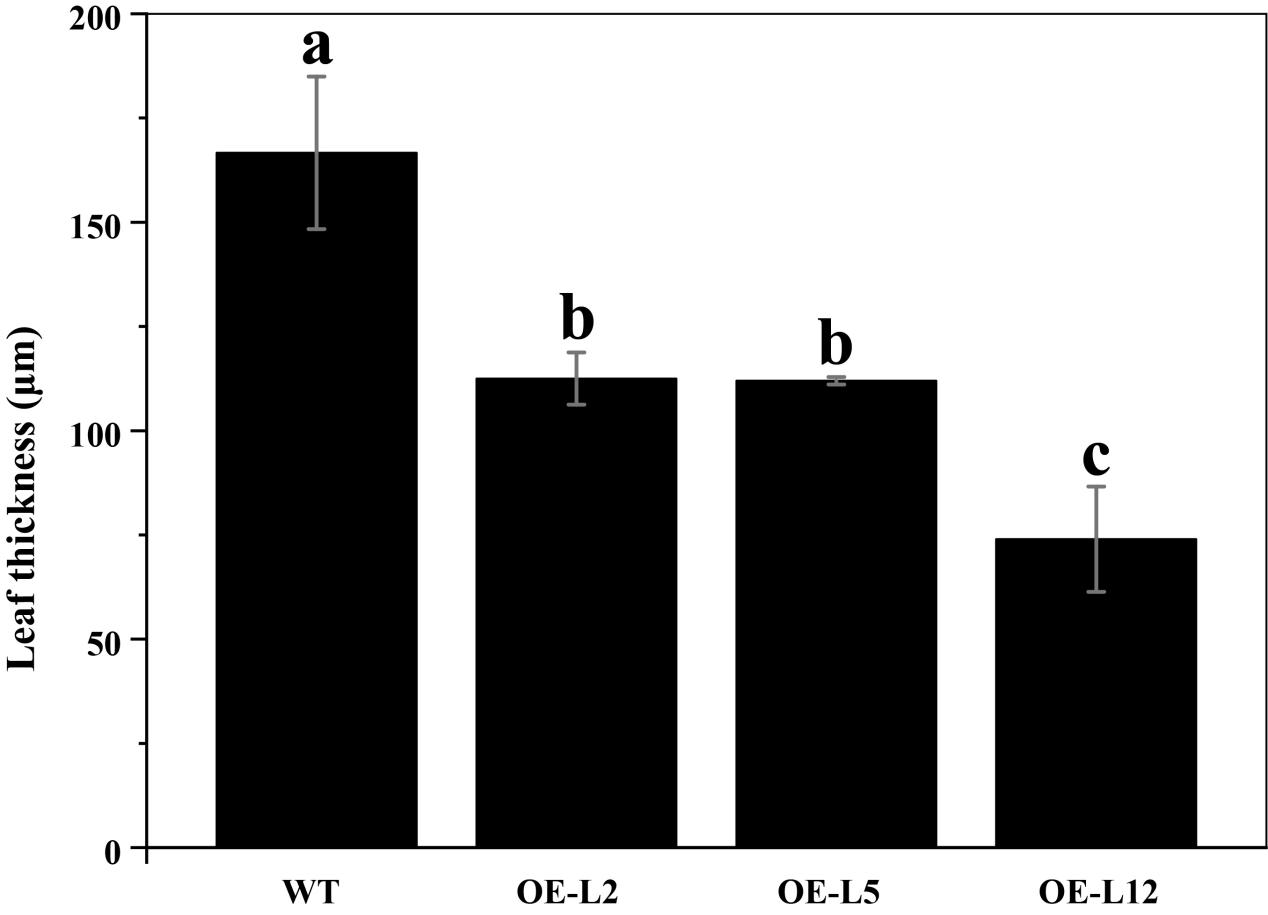 |
| --- |
| **Fig. S6** Leaf thickness of the WT and *OfYABBY12-OE* lines. Error bars indicate the standard deviations of three biological replicates. Different letters above the error bars indicate significant differences at the p < 0.05 level according to Duncan’s multiple range test. |

| **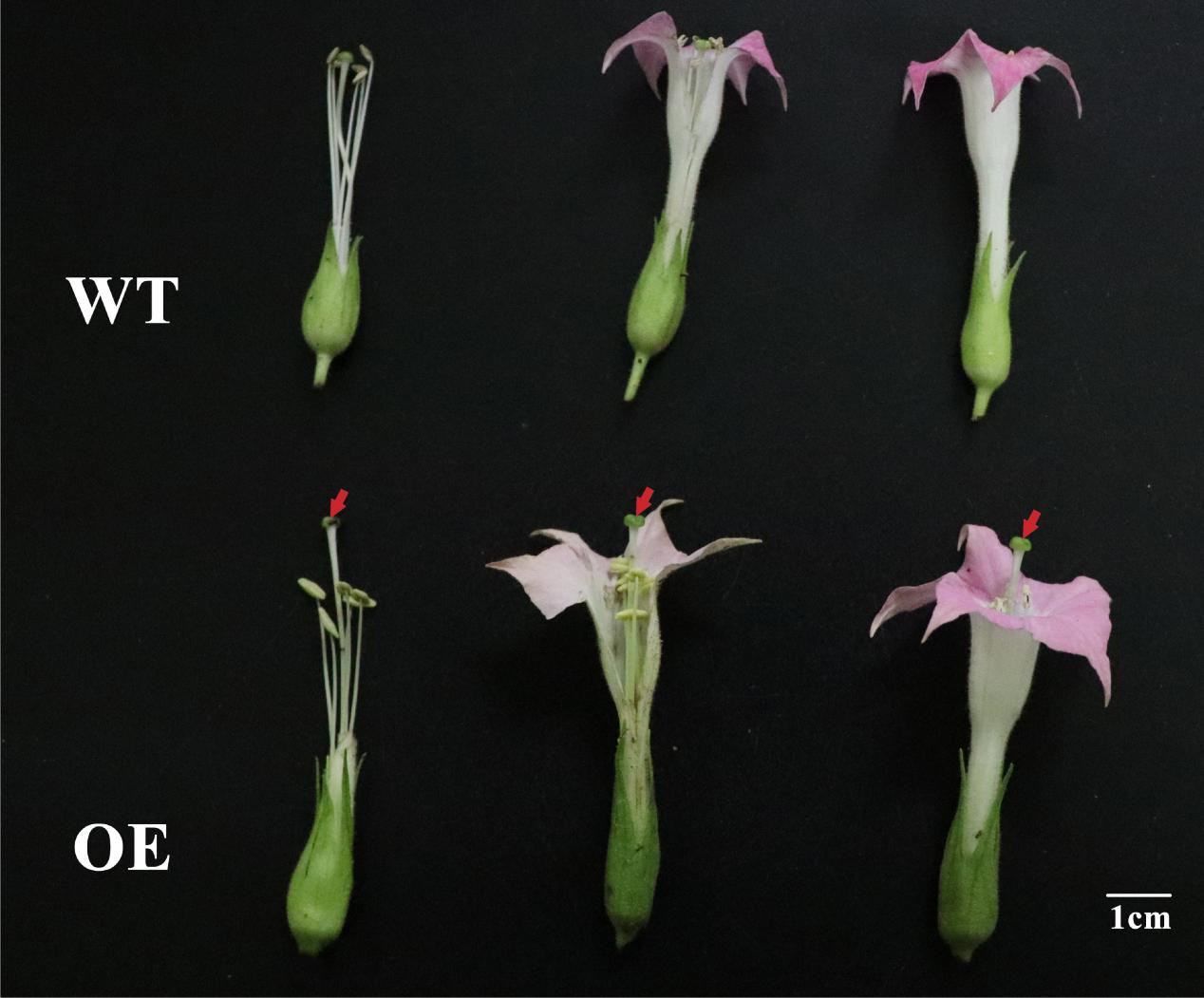** |
| --- |
| **Fig. S7** Stamen and pistil lengths of *OfYABBY12* transgenic plants compared with the WT. |

| 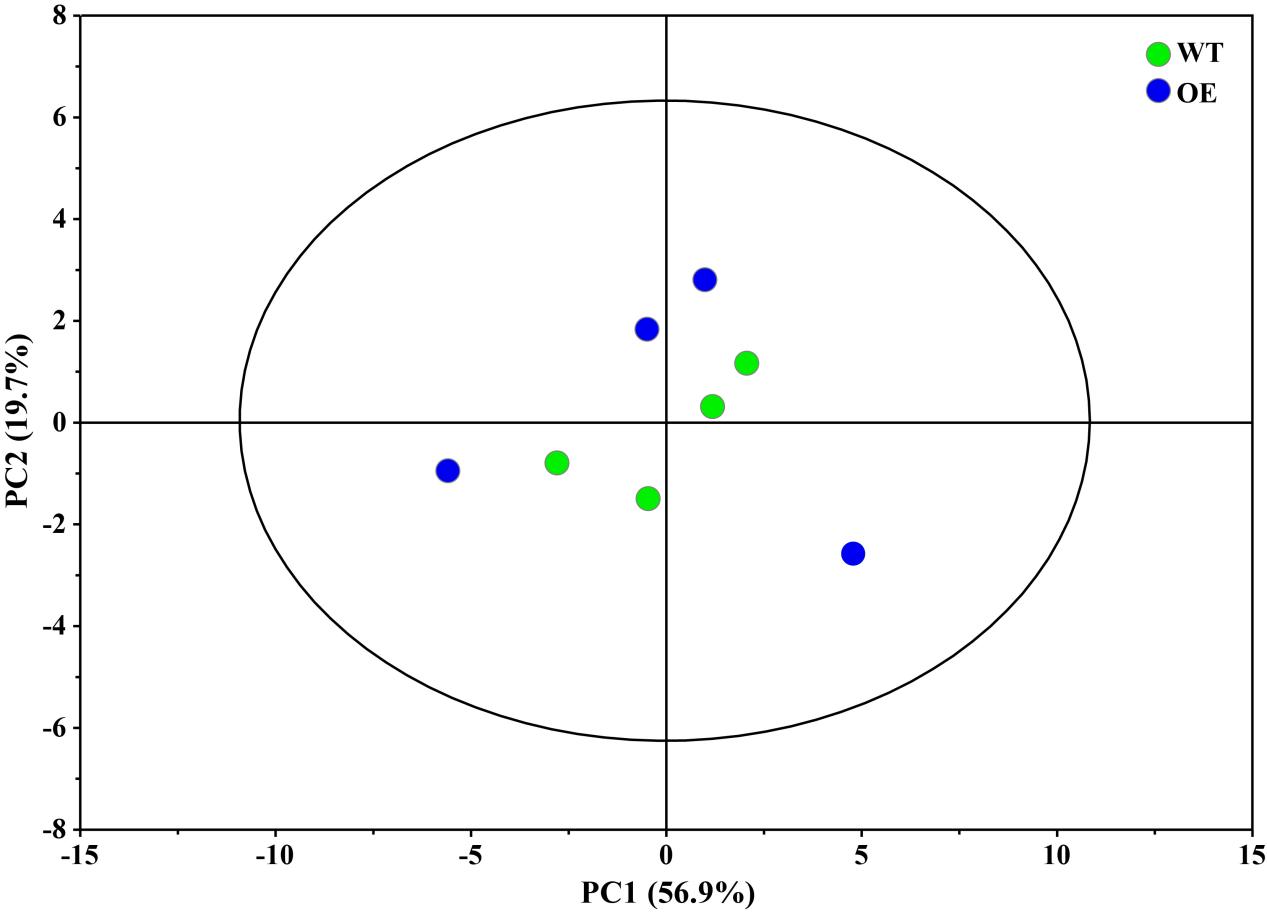 |
| --- |
| **Fig. S8** Principal component analysis (PCA) of VOCs in WT and *OfYABBY12-OE* flowers. |

| 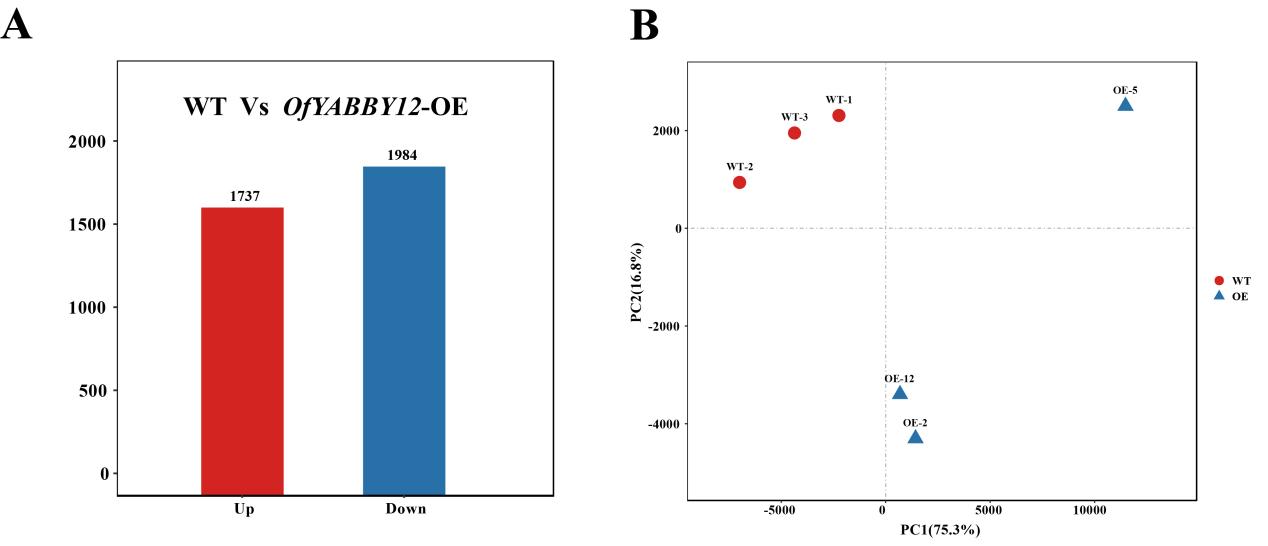 |  |
| --- | --- |
| **Fig. S9** Transcriptome sequencing of tobacco leaves. (A) Number of differentially expressed genes (DEGs) and (B) score scatter plot of the RNA-seq profiles of tobacco leaves with WT and *OfYABBY12-OE* lines. | |

| 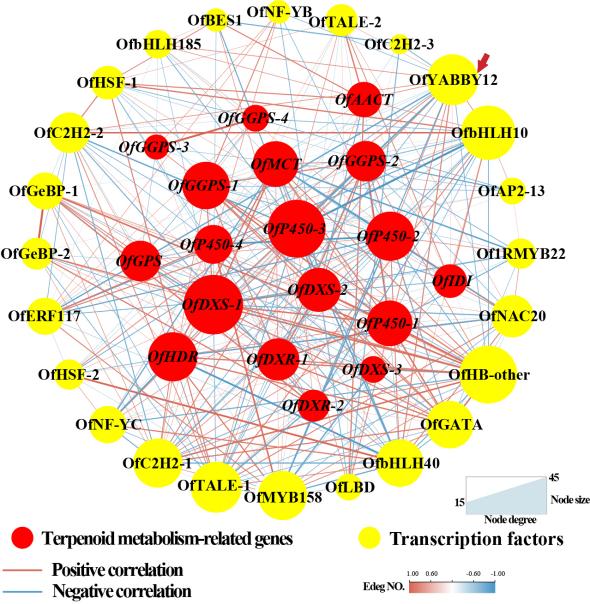 |
| --- |
| **Fig. S10** Molecular regulatory network diagram of β-ionone synthesis. |
